# Supplementary material for: OsbHLHq11, the Basic Helix-Loop-Helix Transcription Factor, Involved in Regulation of Chlorophyll Content in Rice
Source: Biology (Basel). 2022 Jul 1;11(7):1000. doi: 10.3390/biology11071000 (PMC9312294; doi:10.3390/biology11071000)
Supplement: Supplementary file 1 [file biology-11-01000-s001.zip › biology-1792643-supplementary.pdf]

**Table S1.** 26 chlorophyll content-related genes in RM26981-RM287 on chromosome 11.

| Function | Locus | Description |
|---|---|---|
| Transporter | *LOC_Os11g37130* | Twin-arginine translocation protein TatB family protein. |
| | *LOC_Os11g37660* | Protein prenyltransferase domain containing protein. |
| | *LOC_Os11g37700* | Similar to PDR11 ABC transporter. |
| | *LOC_Os11g39020* | Similar to ABCF-type protein. |
| | *LOC_Os11g37330* | Protein prenyltransferase domain containing protein. |
| Signaling | *LOC_Os11g35310* | Phytosulfokine family protein. |
| | *LOC_Os11g38260* | PAP fibrillin family protein. |
| RNA binding | *LOC_Os11g37990* | CRS1/YhbY domain containing protein. |
| | *LOC_Os11g37510* | Similar to 50S ribosomal protein L4. |
| Protein binding | *LOC_Os11g38040* | Thioredoxin domain 2 containing protein. |
| | *LOC_Os11g39140* | Thioredoxin domain 2 containing protein. |
| Nucleotide binding | *LOC_Os11g37260* | Similar to GTP-binding protein-like; root hair defective 3 protein-like. |
| | *LOC_Os11g39540* | Similar to 14-3-3 protein. |
| Kinase | *LOC_Os11g35500* | Protein kinase-like domain containing protein. |
| | *LOC_Os11g36150* | Similar to Receptor kinase-like protein. |
| | *LOC_Os11g36160* | Similar to Receptor kinase-like protein. |
| | *LOC_Os11g36190* | Similar to Receptor kinase-like protein. |
| | *LOC_Os11g36200* | Similar to Receptor kinase-like protein. |
| DNA binding | *LOC_Os11g35320* | BSD domain containing protein. |
| | *LOC_Os11g36030* | HMG-I and HMG-Y, DNA-binding domain containing protein. |
| | *LOC_Os11g38870* | Basic helix-loop-helix dimerisation region bHLH domain containing protein. |
| | *LOC_Os11g39000* | Basic helix-loop-helix dimerisation region bHLH domain containing protein. |
| Catalytic activity | *LOC_Os11g34210* | Similar to Glutamyl-tRNA(Gln) amidotransferase subunit B (EC 6.3.5.-) (Fragment). |
| | *LOC_Os11g35710* | Terpene synthase family protein. |
| | *LOC_Os11g43200* | Similar to Short chain alcohol dehydrogenase-like. |
| | *LOC_Os11g43360* | Similar to Short chain alcohol dehydrogenase-like. |
